# Activation of Epidermal Growth Factor Receptor Is Required for NTHi-Induced NF-κB-Dependent Inflammation

**DOI:** 10.1371/journal.pone.0028216

**Published:** 2011-11-23

**Authors:** Xiangbin Xu, Rachel R. Steere, Christine A. Fedorchuk, Jinjiang Pang, Ji-Yun Lee, Jae Hyang Lim, Haidong Xu, Zhixing K. Pan, Sanjay B. Maggirwar, Jian-Dong Li

**Affiliations:** 1 Department of Microbiology and Immunology, University of Rochester Medical Center, Rochester, New York, United States of America; 2 Aab Cardiovascular Research Institute, University of Rochester Medical Center, Rochester, New York, United States of America; 3 Department of Biology, Center for Inflammation, Immunity and Infection, Georgia State University, Atlanta, Georgia, United States of America; 4 Department of Medical Microbiology and Immunology, University of Toledo Medical Center, Toledo, Ohio, United States of America; University of Minnesota, United States of America

## Abstract

**Background:**

Inflammation is a hallmark of many serious human diseases. Nontypeable *Haemophilus influenzae* (NTHi) is an important human pathogen causing respiratory tract infections in both adults and children. NTHi infections are characterized by inflammation, which is mainly mediated by nuclear transcription factor-kappa B (NF-κB)-dependent production of proinflammatory mediators. Epidermal growth factor receptor (EGFR) has been shown to play important roles in regulating diverse biological processes, including cell growth, differentiation, apoptosis, adhesion, and migration. Its role in regulating NF-κB activation and inflammation, however, remains largely unknown.

**Methodology/Principal Findings:**

In the present study, we demonstrate that EGFR plays a vital role in NTHi-induced NF-κB activation and the subsequent induction of proinflammatory mediators in human middle ear epithelial cells and other cell types. Importantly, we found that AG1478, a specific tyrosine kinase inhibitor of EGFR potently inhibited NTHi-induced inflammatory responses in the middle ears and lungs of mice *in vivo*. Moreover, we found that MKK3/6-p38 and PI3K/Akt signaling pathways are required for mediating EGFR-dependent NF-κB activation and inflammatory responses by NTHi.

**Conclusions/Significance:**

Here, we provide direct evidence that EGFR plays a critical role in mediating NTHi-induced NF-κB activation and inflammation *in vitro* and *in vivo*. Given that EGFR inhibitors have been approved in clinical use for the treatment of cancers, current studies will not only provide novel insights into the molecular mechanisms underlying the regulation of inflammation, but may also lead to the development of novel therapeutic strategies for the treatment of respiratory inflammatory diseases and other inflammatory diseases.

## Introduction

Inflammation is a hallmark of many serious human diseases. Appropriate inflammation is a protective host defense response to remove the injurious stimuli and initiate tissue healing and repair. However, overactive inflammation is detrimental to the host, leading to inflammatory diseases. Thus, inflammation must be tightly regulated. The molecular mechanisms underlying tight regulation of inflammation remain largely unknown. Steroids and cyclooxygenase inhibitors have long been used as the main therapeutic anti-inflammatory agents, but they are frequently associated with significant detrimental effects in patients. In addition, inappropriate antibiotic treatment for bacterial infection contributes significantly to the worldwide emergence of antibiotic resistance. Thus, there is an urgent need for the development of novel anti-inflammatory agents.

Nontypeable *Haemophilus influenzae* (NTHi), a gram-negative bacterium, is an important human pathogen in both children and adults [1]. In children, it causes otitis media (OM), the most common childhood infection and the leading cause of conductive hearing loss [2], [3]. In adults, it exacerbates chronic obstructive pulmonary disease (COPD) [4], [5], an important lung disease and the fourth leading cause of death in the United States [6]. Like most bacterial infections, NTHi infection is characterized by inflammation, which is mainly mediated by nuclear factor-kappa B (NF-κB)-dependent production of proinflammatory mediators [7], [8]. NF-κB is a transcription factor consisting of homo- or heterodimers of Rel-related proteins [9]. It has five members in mammalian cells: RelA (p65), RelB, c-Rel, p50/p105, and p52/p100. The heterodimer consisting of two subunits, p65 and p50, is most commonly involved in the regulation of a variety of physiologic processes, including inflammation, differentiation, proliferation, and survival, among others [9]. In its inactive state, NF-κB resides in the cytoplasm and forms a multiprotein complex with an inhibitory subunit, inhibitor of NF-κB (IκB). Upon activation by external stimuli, the inflammatory signal converges on and activates a set of IκB kinases known as the IκB kinase (IKK) complex, which are composed of three subunits: IKKα, IKKβ, and IKKγ. IκBα is phosphorylated by IKKs and this phosphorylation results in the degradation and dissociation of IκBα from NF-κB. Once released from the complex involving IκBα, NF-κB translocates to the nucleus, where it binds to DNA and promotes the transcription of target genes. NF-κB is activated by inflammatory stimuli and involved in regulating expression of proinflammatory mediators, including cytokines, chemokines, and adhesion molecules, thereby playing a critical role in mediating inflammatory responses [10].

Toll-like receptor 2 (TLR2) plays a crucial role in mediating NTHi-induced inflammatory response. However, directly blocking TLR2 signaling may result in some unwanted detrimental side effects because appropriate immune response mediated by TLR2 signaling is also required for host defense against invading bacterial pathogens. For instance, uncontrolled bacterial growth, decreased bacteria clearance and increased susceptibility to bacterial infection was observed in TLR2 KO mice [11], [12], [13] and impairment of TLR2 signaling due to genetic mutations in human populations closely correlates with increased susceptibility to bacterial pathogens [14], [15]. Thus, identifying a non-TLR2 therapeutic target for NTHi infection is in high demand.

The epidermal growth factor receptor (EGFR) is a member of the HER family composed of four distinct receptors: EGFR/ErbB1, Her-2/ErbB2/c-neu, Her-3/ErbB3, and Her-4/ErbB4, which are predominantly located at the basolateral surface of polarized epithelial cells. EGFR is traditionally known as a growth factor receptor that mediates cell differentiation and proliferation. Elevated levels of EGFR and/or its cognate ligands have been shown to be involved in tumor growth [16]. In addition, EGFR is activated by multiple TLRs to produce innate immune response in airway epithelium [17]. Activation of EGFR plays an important role in recruiting leukocytes [18], inducing mucins and antimicrobial peptides to clear pathogens [19], [20], and increasing wound repair [17]. Recent studies from our group suggested that EGFR is at least in part activated by NTHi via NTHi-derived EGF-like growth factor and plays an important role in negatively regulating TLR2 induction during bacterial infections [21]. In addition, exogenous EGF increases NTHi invasion of host epithelial cells, demonstrating the biological significance of TLR2 regulation by EGFR signaling [21]. However, the role of EGFR in regulating NTHi-induced NF-κB signaling and inflammatory response in airway inflammatory diseases has yet to be fully explored.

Based on the essential role of TLR2 in NTHi-induced NF-κB signaling and inflammatory responses and the role of EGFR in controlling TLR2 induction, we hypothesized that EGFR may regulate NTHi-induced NF-κB activation and inflammation in the middle ear and lung. Here, we provide direct evidence for the critical role of EGFR signaling in regulating NTHi-induced inflammation in human middle ear and airway epithelial cells *in vitro*, and in mouse middle ear and lung *in vivo*. Our studies will not only provide novel insights into the molecular mechanisms underlying the regulation of inflammation, but will also facilitate translational research toward novel therapeutic strategies for the treatment of respiratory and other inflammatory diseases.

## Materials and Methods

### Reagents

AG1478, wortmannin and SB2030580 were purchased from Calbiochem (PA, USA). Polyclonal antibody against phospho-IκBα, IκBα, phospho-IKKα/β, IKKα, IKKβ, phospho-p38, p38, phospho-MKK3/6, MKK3, phospho-Akt, Akt, phospho-EGFR and EGFR were purchased from Cell Signaling (MA, USA). Antibody against actin was purchased from Santa Cruz (CA, USA).

### Bacteria Strain and Culture

Clinical isolate of NTHi wild-type strain 12 was used in *in vitro* cell culture experiments and *in vivo* animal experiments [22], [23]. Bacteria was grown on chocolate agar at 37°C in an atmosphere of 5% CO_2_ overnight and inoculated in brain heart infusion broth supplemented with 3.5 µg of NAD per mL. For *in vitro* experiments, the epithelial cells were treated with NTHi at a multiplicity of infection (MOI) of 1∶25 for various times as indicated in figures. For *in vivo* animal experiments, NTHi was inoculated into the middle ear for the OM model and the lung for the pneumonia model as described below in animal experiments.

### Cell Cultures

Human middle ear epithelial cell line (HMEEC-1) [7], [22], [24], a commonly used middle ear cell line, was derived by human papilloma virus immortalization of primary human middle ear epithelial cells, and was maintained in a 1∶1 mixture of Bronchial Epithelial Basal Medium (BEBM) and Dulbecco's modified Eagle's medium (DMEM) as described [7], [22]. Human airway epithelial cell line (A549), human cervix epithelial cell line (HeLa) and mouse macrophage cell line (RAW 264.7, American Type Culture Collection, Manassas, VA) were maintained as described [22], [25]. MDA-MB453 (hereafter MB453), a breast cancer epithelial cell line in which the level of EGFR expression is not detectable with anti-EGFR antibody, and MDA-MB468 (hereafter MB468), a breast cancer epithelial cell line in which the level of EGFR expression is readily detectable with the same anti-EGFR antibody were maintained as described [21], [26]. All cells were cultured under standard conditions (5% CO_2_ in air in a humidified environment at 37°C).

### Plasmids, Transfections and Luciferase Reporter Assay

The EGFR dominant-negative mutant (DN) and NF-κB-luciferase reporter plasmids were described previously [21], [25]. Cells were co-transfected with NF-κB-luciferase reporter plasmid together with or without EGFR DN expression plasmids. Empty vector was used as a control. All transient transfections were carried out in triplicate using a TransIT-LT1 reagent (Mirus Co.) following the manufacturer's instructions. At 40 hours after the start of transfection, cells were inoculated with NTHi for 5 hours before cell lysis for luciferase assay as described previously.

### RNA-mediated Interference

RNA-mediated interference for down-regulating EGFR expression was carried out using EGFR siRNA as described previously using Lipofectamine 2000 (Invitrogen) [21]. EGFR small interfering RNA oligonucleotide was purchased from Dharmacon. Forty hours after the start of transfection, cells were treated with NTHi for the indicated time before being lysed for luciferase assay.

### RNA Isolation and Real-time Quantitative PCR (Q-PCR)

Total RNA was isolated with TRIzol reagent (Invitrogen) by following the manufacturer's instructions. For the reverse transcription reaction, TaqMan reverse transcription reagents (Applied Biosystems) were used. Briefly, the reverse transcription reaction was performed for 60 min at 37°C, followed by 60 min at 42°C by using oligo (dT) and random hexamers. PCR amplifications were performed by using SYBR Green Universal Master Mix. In brief, reactions were performed in duplicate containing 2X Universal Master Mix, 1 mL of template cDNA and 100 nM primers in a final volume of 12.5 mL, and they were analyzed in a 96-well optical reaction plate (Applied Biosystems). The relative quantities of mRNAs were obtained by using the comparative Ct method and were normalized with pre-developed Taqman assay reagent mouse GAPDH or human cyclophilin as an endogenous control (Applied Biosystems). The primers for human TNF-α, IL-1β, IL-8, cyclophilin, and mouse TNF-α, IL-1β, MIP-2, EGFR, and GAPDH were described previously [25].

### Western Blot Analysis

Cell lysates were prepared in the buffer containing 20 mM Tris-HCl (pH 8.0), 0.5 M NaCl, 0.25% Triton X-100, 1 mM EDTA, 1 mM EGTA, 10 mM glycerophosphate, 10 mM NaF, 300 µM Na_3_VO_4_, 1 mM benzamidine, 2 µM PMSF, 1 mM DTT and protease inhibitor cocktail (Sigma, MO, USA) by scraping, incubating on ice for 30 min, and centrifugation at 12,000 g for 15 min. Supernatant was collected and then subjected to SDS-PAGE, and transferred to poly-vinylidine difluoride membranes. The membrane was blocked with 5% nonfat milk, incubated in a 1∶1,000 dilution of a primary antibody, and incubated with 1∶2,000 dilution of the corresponding secondary antibody. The membrane was reacted with chemiluminescence reagent ECL to visualize the blots.

### Animal Experiments

C57 BL/6 mice were purchased from National Cancer Institute (NCI, NIH), and eight week old male mice were used in this study. For the NTHi-induced OM model, anaesthetized mice were transtympanically inoculated with NTHi under the surgical microscope, and saline was inoculated as control. AG1478 (10 mg/kg of body weight) or an equal volume of vehicle control was administered via an intraperitoneal route 2 hours before the transtympanic inoculation of NTHi. Animals were then sacrificed by intraperitoneal inoculation of 100 mg/kg sodium pentobarbital at 9 and 24 hours after NTHi inoculation. To assess the mRNA expression of proinflammatory mediators, total RNA was extracted from the bullae of NTHi- or saline-inoculated ears at the time points indicated above. For histological analysis, dissected temporal bones were fixed with 10% buffered formaldehyde overnight with rocking, decalcified with CalEX, embedded in paraffin, and sectioned at 5-µM thickness. Sections were then stained with hematoxylin and eosin (H&E) to visualize inflammatory response and pathological changes in the middle ear. H&E-stained middle ear sections were then evaluated using Axiovert 40 CFL (Carl Zeiss), and images were recorded with an AxioCam MRC (Carl Zeiss).

For NTHi-induced pneumonia model, anaesthetized mice were intratracheally inoculated with NTHi, and saline was inoculated as control. AG1478 (10 mg/kg of body weight) or an equal volume of vehicle control was administered via an intraperitoneal route 2 hours before the intratracheal inoculation of NTHi. Animals were then sacrificed by intraperitoneal inoculation of 100 mg/kg sodium pentobarbital at 9 and 24 hours after NTHi inoculation. For histological analysis, dissected lung was inflated and fixed with 10% buffered formaldehyde, embedded in paraffin, and sectioned at 5-µM thickness. Sections were then stained and inspected as described above. For polymorphonuclear neutrophil (PMN) analysis, bronchoalveolar lavage (BAL) was performed by cannulating the trachea with sterilized PBS. Cells from BAL fluid were stained with Hemacolor (EM Science) after cytocentrifugation (Thermo Electronic Co.). To assess the mRNA expression of proinflammatory mediators, total RNA was extracted from the lungs of NTHi- and saline-inoculated mice at the time points indicated above. All animal experiments were approved by the Institutional Animal Care and Use Committee at the University of Rochester (Permission Number: 2007-058 and 2005-209).

### Statistical Analysis

Data are shown as mean±S.D. Statistical evaluation was done by unpaired Student's *t* test and *p*<0.05 was taken as a significant difference.

## Results

### EGFR plays a critical role in mediating NTHi-induced NF-κB activation and subsequent inflammatory response *in vitro*

**Figure 1:**
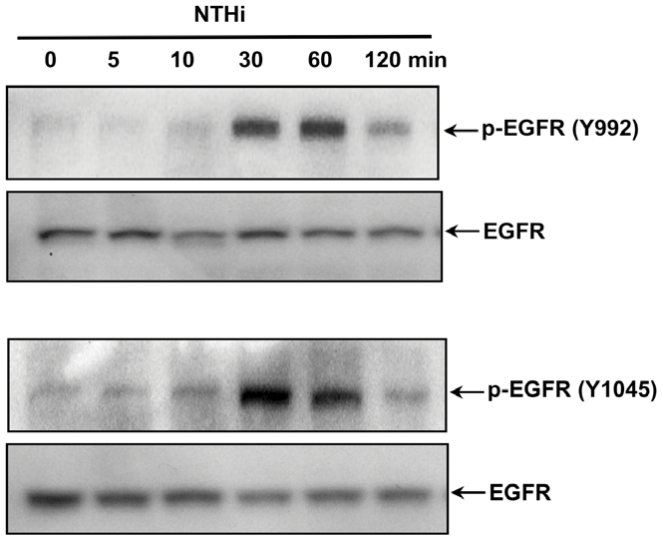
EGFR is activated by NTHi in middle ear epithelial cells. HMEEC-1 cells were treated with NTHi for the time indicated, then were lysed and blotted with anti-phospho-EGFR and EGFR antibody. Data are representative of three or more independent experiments.

EGFR represents one of the important tyrosine kinases and can be activated via phosphorylation by many stimuli. We first evaluated if EGFR is phosphorylated by NTHi. As shown in Fig 1, NTHi induced EGFR phosphorylation in a time-dependent manner in HMEEC-1 cells.

**Figure 2:**
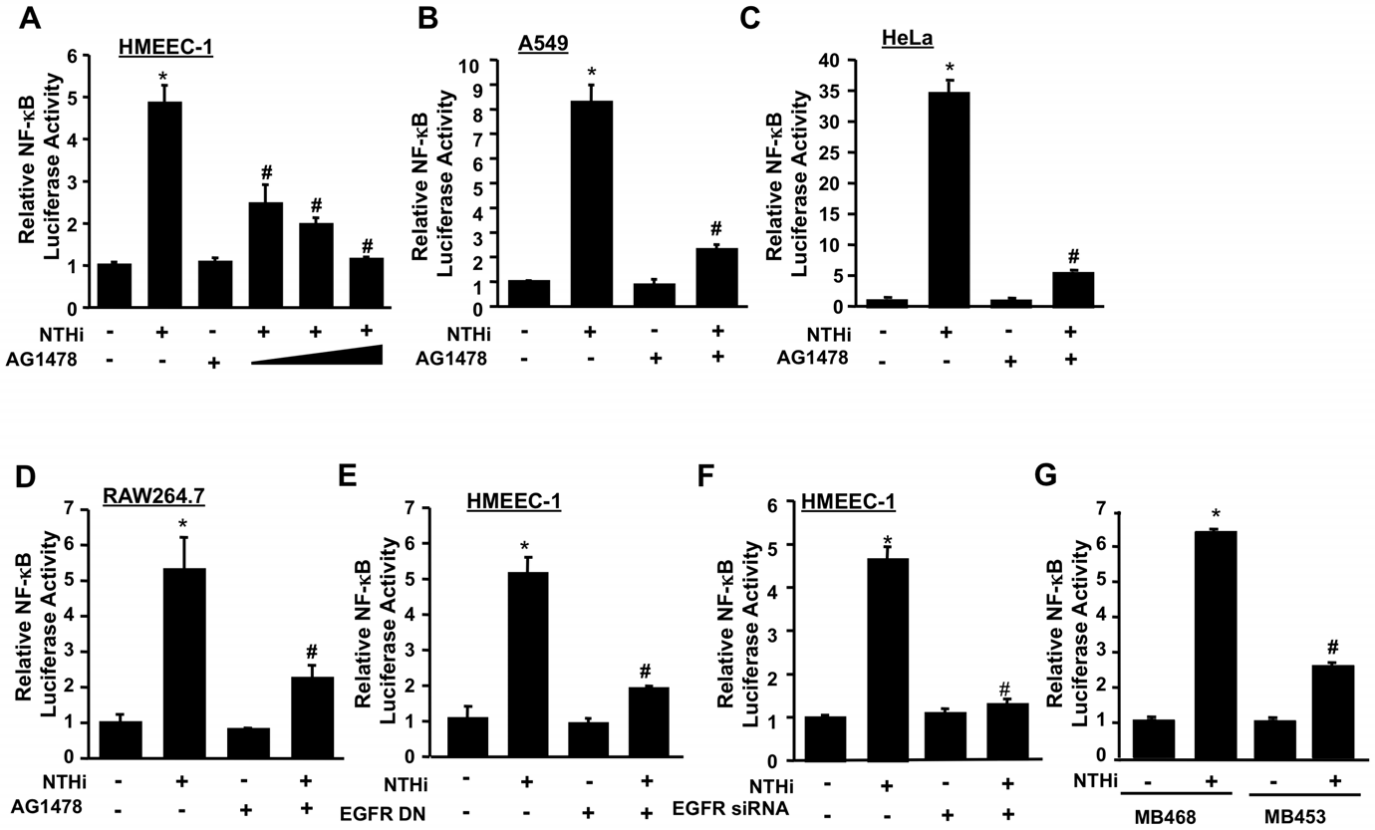
EGFR mediates NTHi-induced NF-κB transcriptional activity in a variety of cell types. (*A*–*D*) The effect of AG1478 on NTHi-induced NF-κB activation was evaluated by performing luciferase assay in HMEEC-1 (A), A549 (B), HeLa (C) and RAW 264.7(D). (*E*–*F*) Overexpression of EGFR DN (E) or EGFR knockdown using EGFR siRNA (F), and NTHi-induced NF-κB activation was measured by performing luciferase assay. (*G*) NTHi-induced NF-κB activation was measured by luciferase assay in EGFR-competent MB468 and EGFR-deficient MB453 cells. Data represent the mean±SD of at least three independent experiments, and each experiment was performed in triplicate. *p <0.05 vs. control; #*p* <0.05 vs. NTHi alone.

Because NTHi infection is mainly characterized by inflammation, it is likely that EGFR may play an important role in mediating NTHi-induced inflammatory response, which is mainly mediated by NF-κB. We thus determined if EGFR is involved in NTHi-induced NF-κB activation by using multiple approaches to assess the effects on NTHi-induced NF-κB activation of AG1478 (a chemical inhibitor for EGFR), EGFR DN, EGFR siRNA, and EGFR-deficient MB-453 cells. As shown in Fig. 2, NTHi greatly induces NF-κB activation in HMEEC-1, A549, HeLa, RAW 264.7 and MB-468 cells. Interestingly, AG1478 markedly inhibited NTHi-induced NF-κB activation in a dose-dependent manner (Fig. 2A–D). Please note that no significant effect of AG1478 on cell morphology and viability was observed at the concentration used in the experiments (data not shown). Moreover, overexpression of EGFR DN (Fig. 2E) and knockdown of EGFR using EGFR siRNA (Fig. 2F) greatly inhibited the NTHi-induced NF-κB activation. Furthermore, NTHi-induced NF-κB luciferase activity was much lower in MB453 cells compared to that in MB468 cells (Fig. 2G). Taken together, these data indicate that EGFR plays a critical role in mediating NTHi-induced NF-κB activation *in vitro*.

**Figure 3:**
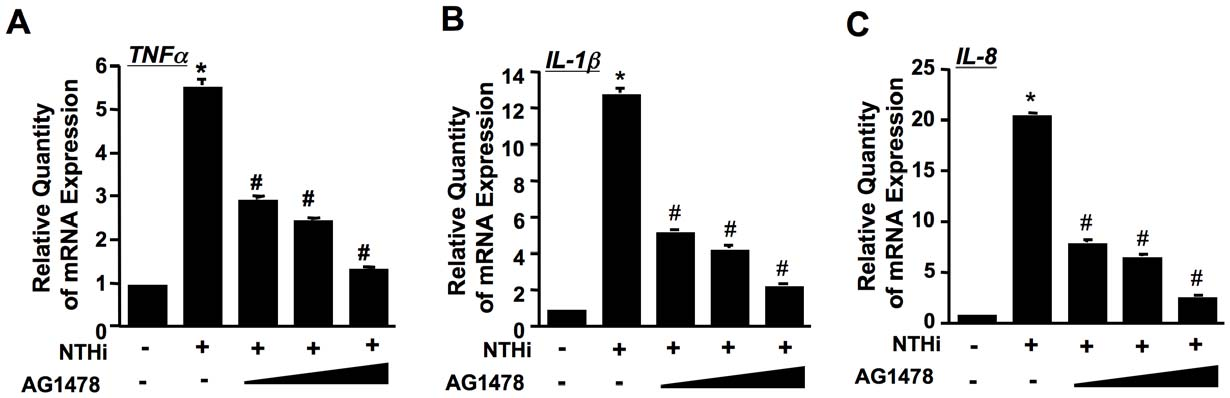
EGFR mediates NTHi-induced upregulation of proinflammatory mediators in middle ear epithelial cells. (*A*–*C*) Cells were treated with EGFR inhibitor AG1478 for 1 hour followed by 5 hour NTHi treatment. The mRNA expression of TNF-α, IL-1β and IL-8 was measured by performing Q-PCR analysis. Data represent the mean ± SD of at least three independent experiments, and each experiment was performed in triplicate. *p<0.05 vs. control; #*p*<0.05 vs. NTHi alone.

We further determined the role of EGFR in mediating NTHi-induced NF-κB-dependent up-regulation of proinflammatory mediators. As shown in Fig. 3A–C, AG1478 greatly inhibited NTHi–induced up-regulation of TNF-α, IL-1β and IL-8 mRNA in a dose-dependent manner, thereby demonstrating the critical role for EGFR in NTHi-induced pro-inflammatory responses in epithelial cells.

### EGFR is also crucial for mediating NTHi-induced inflammation in the middle ear and lung tissues of mice *in vivo*

**Figure 4:**
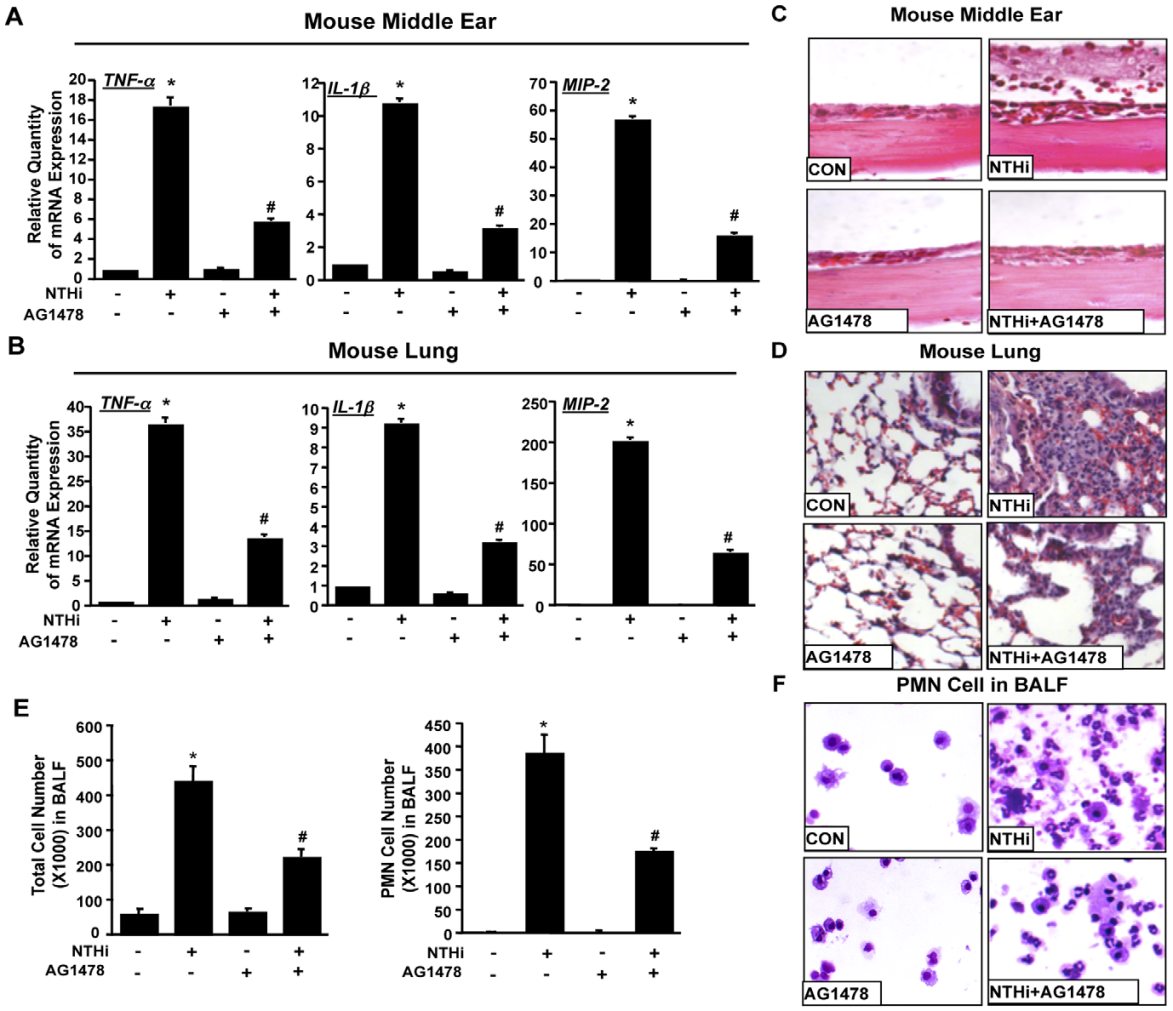
Inhibition of EGFR inhibited NTHi-induced inflammation in the middle ear and lung tissues of mice *in vivo*. *(A & B)* Animals were intraperitoneally inoculated with AG1478 or vehicle control. Two hours after AG1478 inoculation, NTHi was inoculated into middle ear via tympanic membrane (A) or into trachea (B). The mRNA expression of TNF-α, IL-1β, and MIP-2 was measured in the middle ear (A) or lung tissues (B) of mice inoculated with NTHi or saline as a control. *p<0.05 vs. untreated group; #*p*<0.05 vs. NTHi alone. (*C & D*) Middle ear (C) and lung tissues (D) of mice inoculated with NTHi with or without AG1478 were stained with Hematoxylin and Eosin for histological analysis (H&E stain, magnification ×200). *(E & F*) Bronchoalveolar lavage (BAL) was performed in NTHi-inoculated mice with or without AG1478, and total and polymorphonuclear (PMN) neutrophils were counted (E) and cytocentrifuged to stain with Hemacolor (F).

To further confirm the involvement of EGFR in NTHi-induced inflammation, we determined if EGFR inhibitor AG1478 inhibits inflammation in the middle ear and lung tissues of mice *in vivo*. As shown in Fig. 4A and 4B, intraperitoneal administration of AG1478 significantly inhibited mRNA expression of TNF-α, IL-1β, and MIP-2 in the ears and lungs of mice after transtympanic or intratracheal inoculation of NTHi. Consistent with this result, histological analysis of the ear and lung tissues of NTHi-inoculated mice showed that AG1478 markedly inhibited leukocyte infiltration (Fig. 4C and 4D). Also in agreement with these results, AG1478 significantly inhibited polymorphonuclear neutrophil (PMN) infiltration in bronchoalveolar lavage (BAL) fluids (Fig. 4E and 4F). These results demonstrate that EGFR is critical for mediating NTHi-induced inflammatory responses *in vivo*.

### EGFR mediates NTHi-induced NF-κB activation via an IKKα/β-IκBα-dependent pathway in middle ear epithelial cells

**Figure 5:**
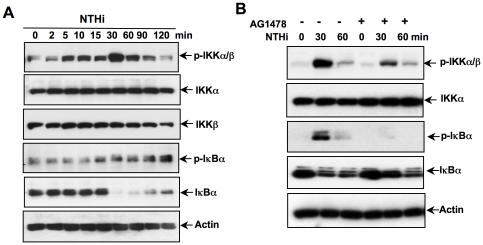
EGFR mediates NTHi-induced NF-κB activation by inducing IKKα/β and IκBα activation in middle ear epithelial cells. (*A*) Cells were treated with NTHi at various time points as indicated in the figure, and whole cell protein was collected and blotted against total- and phospho-IKKα/β and IκBα. (*B*) Cells were treated with NTHi with or without AG1478, and whole cell protein was collected and blotted against total- and phospho-IKKα/β and IκBα. Data are representative of three or more independent experiments.

IKKα/β plays a key role in NTHi-induced NF-κB activation by inducing phosphorylation of IκBα. Thus, we determined whether EGFR mediates NTHi-induced NF-κB activation by regulating the IKKα/β-IκBα pathway. As shown in Fig. 5A, NTHi induced phosphorylation of IKKα/β and IκBα in a time-dependent manner. Furthermore, inhibition of EGFR using AG1478 significantly inhibited NTHi-induced IKKα/β and IκBα phosphorylation (Fig. 5B). These data indicate that EGFR acts upstream of IKKα/β in mediating NTHi-induced NF-κB activation.

### MKK3/6-p38 MAPK also mediates EGFR-dependent inflammation induced by NTHi in middle ear epithelial cells

**Figure 6:**
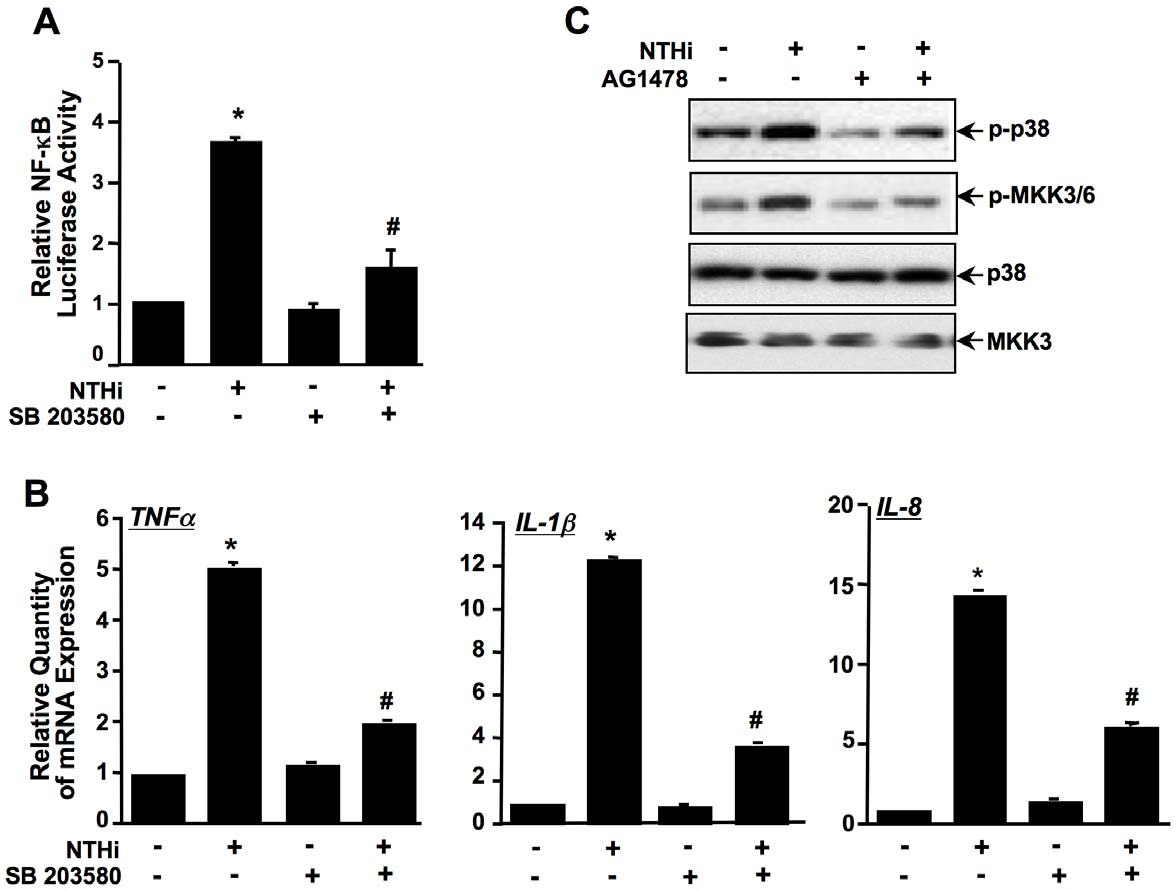
MKK3/6-p38 mediates EGFR-dependent inflammation induced by NTHi in middle ear epithelial cells. (*A*) Cells were incubated with NTHi with or without SB203580, and NTHi-induced NF-κB activation was measured by performing luciferase assay. (*B*) Cells were incubated with NTHi with or without SB203580, and mRNA expression of TNF-α, IL-1β and IL-8 was measured by performing Q-PCR analysis. (*C*) Cells were incubated with NTHi with or without AG1478, and whole cell protein was collected and blotted against total- and phospho-p38 and MKK3/6. Data represent the mean±SD of at least three independent experiments, and each experiment was performed in triplicate. *p<0.05 vs. control; #*p*<0.05 vs. NTHi alone.

Having demonstrated that EGFR mediates NTHi-induced NF-κB activation and the subsequent inflammatory responses, we sought to determine which signaling pathways are involved in EGFR-mediated NF-κB-dependent inflammatory responses induced by NTHi. The p38 mitogen-activated protein kinase (MAPK) signaling pathway plays an important role in NTHi-induced NF-κB activation [22] and has also been found to be important in the EGFR signaling pathway [27]. We thus determined if p38 MAPK is involved in EGFR-mediated NF-κB activation and inflammatory responses induced by NTHi. As shown in Fig. 6, p38-specific inhibitor SB203580 inhibited NTHi-induced NF-κB activation (Fig. 6A) and also mRNA expression of proinflammatory mediators (TNF-α, IL-1β and IL-8) (Fig. 6B) in HMEEC-1. It should be noted that SB203580 exhibited no effect on NTHi-induced IKKα/β and IKBα phosphorylation and nuclear translocation of NF-κB, thereby indicating that p38 mediates NTHi-induced NF-κB activation independently of IKKα/β and IKBα as well as nuclear translocation of NF-κB (data not shown). Next, we determined if EGFR is involved in NTHi-induced activation of MKK3/6 and p38 MAPK by assessing the effect of EGFR inhibitor on NTHi-induced MKK3/6 and p38 phosphorylation. As shown in Fig. 6C, AG1478 inhibited NTHi-induced MKK3/6 and p38 phosphorylation in HMEEC-1 cells. Together, these data suggest that the MKK3/6-p38 signaling pathway also mediates EGFR-dependent NF-κB activation and inflammatory responses induced by NTHi.

### PI3K/Akt mediates EGFR-dependent inflammatory response induced by NTHi in middle ear epithelial cells

**Figure 7:**
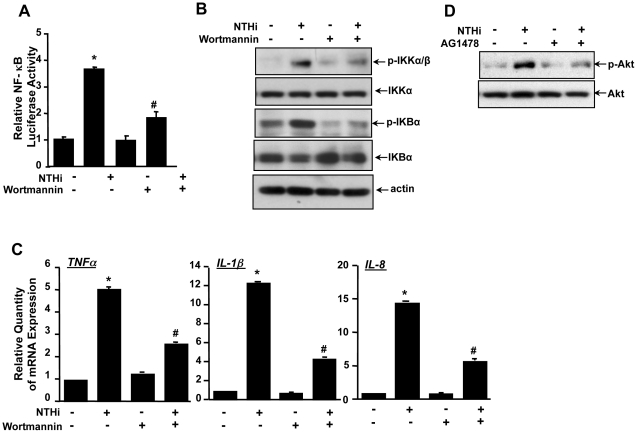
PI3K/Akt mediates EGFR-dependent inflammation induced by NTHi in middle ear epithelial cells. *(A)* Cells were incubated with NTHi with or without wortmannin, and NTHi-induced NF-κB activation was measured by performing luciferase assay. *(B)* Cells were incubated with NTHi with or without wortmannin, and whole cell protein was collected and blotted against total- and phospho-IKKα/β and IκBα. *(C)* Cells were incubated with NTHi with or without wortmannin, and mRNA expression of TNF-α, IL-1β and IL-8 was measured by performing Q-PCR analysis. (*D*) Cells were incubated with NTHi with or without AG1478, and whole cell protein was collected and blotted against total- and phospho-Akt. Data represent the mean±SD of at least three independent experiments, and each experiment was performed in triplicate. *p<0.05 vs. control; #*p*<0.05 vs. NTHi alone.

Previously it has been reported that that EGFR acts as the major upstream activator of phosphatidylinositol 3-kinase (PI3K)/Akt pathway leading to activation of NF-κB [28]. Thus, we determined if PI3K/Akt signaling is also involved in EGFR-dependent NF-κB activation and inflammatory responses in middle ear epithelial cells [28]. As shown in Fig. 7A and B, wortmannin, a specific inhibitor for PI3K, inhibited NTHi-induced NF-κB luciferase activity (Fig. 7A) and phosphorylation of IKKα/β and IκBα (Fig. 7B). Consistent with these findings, wortmannin inhibited NTHi-induced mRNA expression of TNF-α, IL-1β and IL-8 (Fig. 7C) in HMEEC-1, thereby indicating that the PI3K/Akt signaling pathway mediates NTHi-induced NF-κB activation and inflammatory response. We next determined if EGFR acts upstream of PI3K/Akt by assessing the effect of AG1478 on NTHi-induced Akt phosphorylation. As shown in Fig. 7D, AG1478 inhibited NTHi-induced Akt phosphorylation. Collectively, these data demonstrate that PI3K/Akt signaling mediates EGFR-dependent NF-κB activation and inflammatory response induced by NTHi.

## Discussion

**Figure 8:**
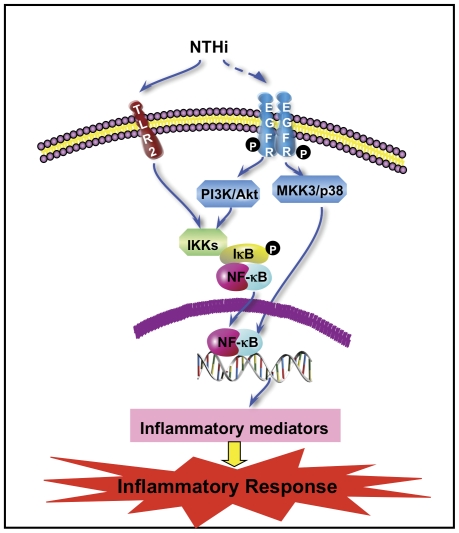
Schematic representation of EGFR-mediated inflammation. As indicated, EGFR is activated by NTHi, and mediates NTHi-induced NF-κB activation and inflammation via MKK3/6-p38 and PI3K/Akt signaling pathways.

In the present study, we provide direct evidence that EGFR mediates NTHi-induced NF-κB activation and subsequent inflammation *in vitro* and *in vivo*. Importantly, we demonstrate that AG1478, the specific tyrosine kinase inhibitor of EGFR, potently inhibited NTHi-induced inflammatory responses in the middle ears and lungs of mice. Moreover, we found that MKK3/6-p38 and PI3K/Akt signaling pathways mediate EGFR-dependent NF-κB activation and subsequent inflammatory responses induced by NTHi (Fig. 8). p38, an important MAPK family member, is activated by multiple stimuli including bacteria such as NTHi, cytokines (IL-1, TNF-α) and growth factors [29]. The activated p38 mediates a variety of cellular responses including inflammation [29]. Its major upstream kinases include MKK3 and MKK6. MKK3/6-p38 pathway plays an important role in NTHi-induced NF-κB activation and mucin production [22]. In addition, p38 is actively involved in mediating EGFR signaling [21], [27], [30]. Our data indicate that MKK3/6-p38 mediates EGFR-dependent NF-κB activation and inflammation by NTHi in middle ear and lung tissues of mice by involving nuclear events of NF-κB signaling. PI3K facilitates a broad range of cellular functions in response to extracellular signals. A key downstream effector of PI3K is the serine-threonine kinase Akt. There is evidence that EGFR acts as the major upstream activator of the PI3K/Akt pathway leading to activation of NF-κB in PC-3 cells [28]. Moreover, NTHi activates the PI3K/Akt pathway in epithelial cells [31]. Our data suggest that PI3K/Akt may act as another important signaling transducer mediating EGFR-dependent NF-κB activation and inflammation induced by NTHi in middle ear and lung inflammation.

Of particular interest in this study is the identification of EGFR as a critical mediator in NTHi-induced NF-κB activation and inflammation *in vitro* and *in vivo*. Mutations, amplifications or misregulations of EGFR or family members are implicated in about 30% of all epithelial cancers. Many anticancer therapeutic approaches are aimed at EGFR. It has also been reported that EGFR plays an important role in the pathogenesis of asthma [32], [33], [34], [35] and inhibitors of tyrosine kinase have been studied as a novel therapeutic strategy for the treatment of asthma [36]. Recent studies suggested that EGFR might also play an important role in inflammation [37], [38], [39], and Gefinitib, a clinically approved EGFR inhibitor, has been used for allergic airway inflammation [40]. We previously showed that NTHi, at least in part, induces EGFR signaling likely via NTHi-derived EGF-like factor although our data do not preclude the involvement of other mechanism [21]. TLRs are critical for detecting invading microbial pathogens by recognizing pathogen associated molecular patterns (PAMPs). Among many TLRs, TLR2 detects NTHi and activates downstream signaling pathways to induce inflammatory responses against NTHi. The important role of TLR2 in bacterial clearance has been reported in both animal studies and human clinical studies. For instance, deficiency of TLR2 resulted in uncontrolled bacterial growth and increased susceptibility to bacterial infection in TLR2 KO mice. Moreover, impairment of TLR2 signaling due to genetic mutations in human populations closely correlated with increased susceptibility to bacterial pathogens. In our previous study, we found that NTHi not only activates the TLR2 signaling pathway, but also induces TLR2 expression, which may further enhance NTHi clearance dependent on TLR2-mediated inflammatory responses. Besides TLR2, NTHi also activates EGFR, which negatively regulates TLR2 expression as evidenced by the finding that inhibition of EGFR using AG1478 enhanced NTHi-induced TLR2 expression. Based on the finding showing that exogenous EGF enhanced NTHi invasion and survival in epithelial cells, EGFR-mediated inhibition of TLR2 expression may exemplify the subversion of the host signaling pathway by bacteria, as NTHi promotes bacterial survival in the host by inhibiting TLR2-mediated antibacterial inflammatory response. Thus, enhancing TLR2 expression by inhibiting EGFR using AG1478 may provide a benefit to the host as it promotes NTHi clearance. However, it should be noted that uncontrolled TLR2 expression may also result in unwanted excess inflammatory responses in vivo. In the present study, we found that EGFR mediates NTHi-induced NF-κB activation and subsequent inflammatory responses because inhibition of EGFR via AG1478 inhibits NTHi-induced NF-κB activation and subsequent inflammatory responses. This finding is of particular interest because EGFR inhibition using AG1478 will promote the host's ability to detect invading pathogens by enhancing TLR2 expression; on the other hand, AG1478 will also inhibit NF-κB activation and subsequent inflammatory responses and thus prevent the uncontrolled inflammatory response that is caused by enhanced expression of TLR2. Taking advantage of clinically available EGFR inhibitors such as gefitinib, lapantinib, erlotinib, cetuximab and panitumumab, findings from this study may not only unveil novel mechanisms underlying the regulation of inflammation, but may also facilitate translational research toward developing novel therapeutic strategies for the treatment of respiratory and other inflammatory diseases.
